# AST/ALT ratio as a significant predictor of the incidence risk of prostate cancer

**DOI:** 10.1002/cam4.3086

**Published:** 2020-06-20

**Authors:** Jiatong Zhou, Zhaowei He, Shenfei Ma, Ranlu Liu

**Affiliations:** ^1^ Department of Urology The Second Hospital of Tianjin Medical University Tianjin China

**Keywords:** AST/ALT ratio, benign prostatic hyperplasia, prostate cancer

## Abstract

**Background:**

To investigate the effect of serum aspartate transaminase/alanine transaminase (AST/ALT) on the risk of prostate cancer.

**Methods:**

A total of 404 patients undergoing prostate biopsy from April 2016 to July 2019 were enrolled. One hundred and ninety‐four patients with prostatic cancer (PCa) were diagnosed by pathology. Two hundred and ten patients were diagnosed with benign prostatic hyperplasia (BPH). Multivariate logistic regression was used to analyze the effect of AST/ALT ratio and other factors on the incidence of PCa.

**Result:**

AST/ALT ratio was significantly higher in PCa than in BPH patients (OR 2.313, 95%CI 1.337‐4.003, *P* = .002). ROC curve indicated that the best cutoff was 1.155 in predicting the incidence risk of PCa. The age of PCa patients is higher than BPH patients (OR 1.054, 95%CI 1.027‐1.082, *P* < .001). We also found that platelets were lower in PCa than in BPH patients. Multivariate analysis showed that AST/ALT ratio could be used as an independent predictor to assess the incident risk of PCa(OR 1.043, 95%CI 1.014‐1.072, *P* = .003). However, AST/ALT ratio did not predict the incidence in high‐risk or low‐risk PCa.

**Conclusion:**

AST/ALT ratio was an independent factor in predicting the incidence of PCa. When the level of AST/ALT ratio in serum raised, the incidence risk of PCa was significantly increased, which was helpful for the clinical diagnosis of PCa. We still needed a multicenter study to verify the role of AST/ALT ratio in the development of PCa.

## Graphical abstract

To investigate the effect of serum aspartate transaminase/alanine transaminase (AST/ALT) on the risk of prostate cancer. AST/ALT ratio was an independent risk factor in predicting the incidence of PCa.

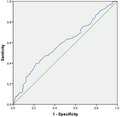

## 1 INTRODUCTION

Background Prostatic cancer (PCa) is one of the most common urinary tumors in the male population, and in 2016 statistics, the incidence of PCa in American men is still high.^1^ In recent years, due to the incidence of PCa in China rise, we need to find more abundant biochemical indicators to better predict the occurrence and development of PCa. Aspartate aminotransferase (AST) and alanine aminotransferase (ALT) are the major circulating enzymes in the serum. Their serum levels not only indicate liver cell damage and death but also serve as predictors in predicting several malignant tumors such as pancreatic cancer and breast cancer.^2^, ^3^ The concept of aspartate transaminase/alanine transaminase (AST/ALT) was proposed in 1957 for the study of hepatitis etiology.^4^ This concept had been proposed so far, the AST/ALT ratio has evolved to be associated with other nonliver diseases such as type 2 diabetes, peripheral vascular disease, acute stroke, and esophageal cancer.^5^, ^6^, ^7^, ^8^ However, there are currently few studies on the relationship between AST/ALT ratio in predicting the occurrence of PCa. Therefore, in order to investigate the impact of AST/ALT ratio on PCa, we performed this study.

## 2 METHODS

### 2.1 Patients enrollment

In this study, 404 patients who underwent prostate biopsy from Tianjin Medical University Second Hospital from April 2016 to July 2019 were enrolled. One hundred and ninety‐four patients with PCa were diagnosed by pathology. Two hundred and one patients with Benign prostatic hyperplasia (BPH). Two hundred and forty‐nine patients with serum total PSA (tPSA) levels ≥ 10 ng/mL, and 155 patients with tPSA levels <10 ng/mL.

Inclusion criteria: (a) All underwent ultrasound‐guided prostate biopsy; (b) Pathological examination confirmed PCa or benign prostate lesions. (c) Exclusion criteria: (a) Excluding patients with chronic prostatitis, acute prostatitis or urinary tract infection; (b) admission to tPSA < 4 ng/mL, (c) patients with previous history of liver disease.

### 2.2 Evaluation

Blood samples (including liver function, complete blood counts) were collected 1‐7 days before surgery. PSA value before biopsy was collected in this study. The upper limit of the reference range, in consideration, was 40 IU/L for AST and 56 IU/L for ALT.

### 2.3 Statistical analysis

The statistical software package SPSS for Windows, version 22, was used to perform statistical analyses. Measurement data were expressed as $\bar{x}$ ± s, and the comparison method between groups was analyzed by analysis of variance. We investigated the potential effect modification of these associations by disease aggressiveness at diagnosis, where “high‐risk PCa” was defined as having a Gleason score ≥ 8, or PSA >20 ng/mL, or tumor stage ≥ T2c. Using these criteria, correlation analysis was performed by multivariate logistic regression. All *P* values were two‐sided, and those < .05 were considered to be statistically significant.

## 3 RESULTS

**Figure 1:**
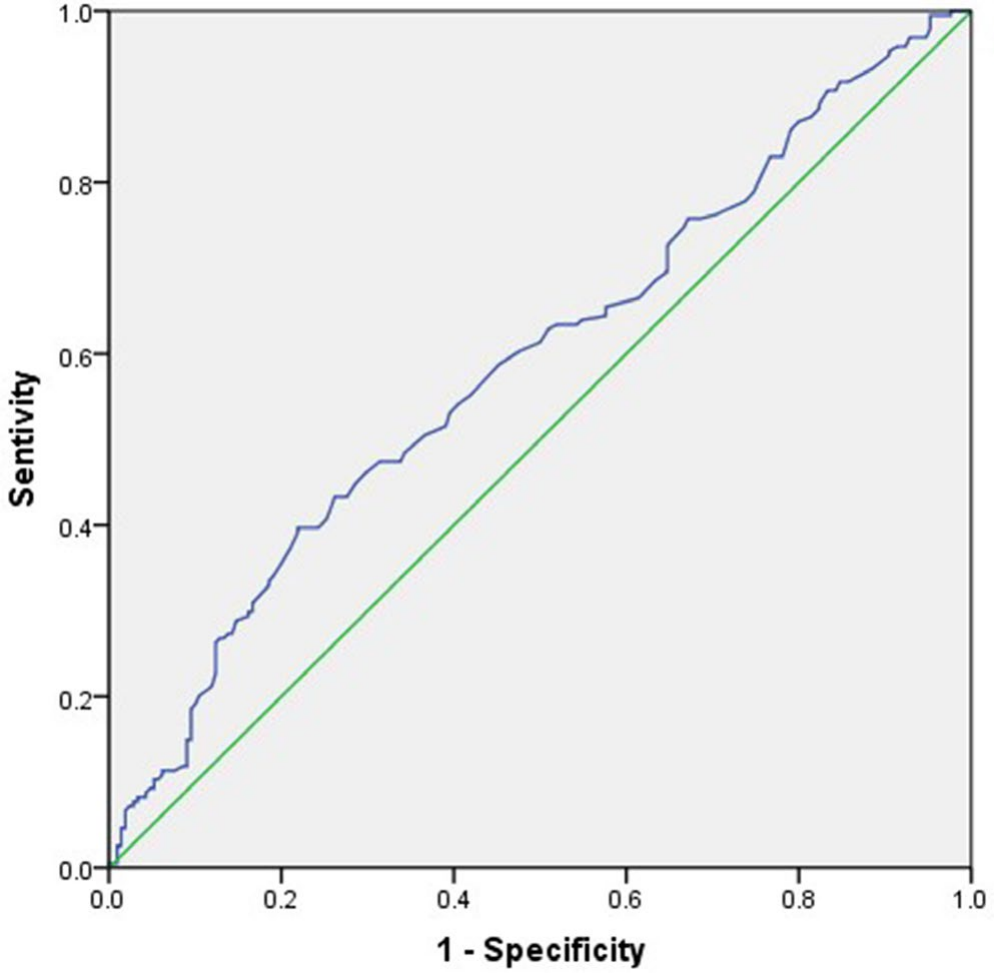
ROC curves in predicting biochemical recurrence by AST/ALT ratio (continuous, AUC = 0.59). ROC receiver operating characteristic, AUC area under curve

The mean age in PCa patients was 69.79 years, and in BPH patients was 66.64 years. The mean AST and ALT value was 20.09 and 20.08 IU/L in PCa patients, and the mean AST and ALT value in BPH patients was 19.37 and 21.71 IU/L. The mean AST/ALT ratio was 1.13 in PCa patients and 1.00 in BPH patients. ROC curve indicated that a AST/ALT ratio of 1.155 had maximum Youden index value (Figure 1) (sensitivity = 39.7%, Specificity = 78.4%); thus, patients with AST/ALT ratio < 1.155 had low risk of PCa, while patients with AST/ALT ratio > 1.155 had high risk of incident PCa. The comparison of clinical and pathological characteristics between PCa patients and BPH patients are shown in Table 1. We found that the age of the patients with PCa was higher than the patients with BPH (OR 1.054, 95%CI 1.027‐1.082, *P* < .001), while the platelet level of the patients with BPH was higher than that PCa patients (OR 0.996, 95%CI 0.993‐1, *P* = .024), and we thought that the AST/ALT ratio in the patients with PCa was significantly higher than in BPH patients (OR 2.313, 95%CI 1.337‐4.003, *P* = .002). However, we did not find significant effects of hypertension, diabetes, cardiovascular disease on the risk of PCa. There was no significant difference in neutrophil/lymphocyte ratio (NLR) between PCa and BPH patients.

**Table 1:** Clinical and pathological characteristics of PCa and BPH

	PCa (n = 194)	BPH(n = 210)	*P* value
Age (y) mean ± *SD*	69.79 ± 8.24	66.64 ± 7.29	<.001*
BMI, mean ± *SD*	24.71 ± 3.96	24.27 ± 2.98	.182
Diabetes, no. (%)			.773
Absent	170(87.6)	182(86.7)	
Present	24(12.4)	28(13.3)	
Hypertension, no. (%)			.687
Absent	107(54.9)	120(57.14)	
Present	87(45.1)	90(42.86)	
Coronary heart disease, no. (%)			.107
Absent	158(79.8)	181(86.19)	
Present	39(20.2)	29(13.81)	
NLR, mean ± SD	2.60 ± 1.46	2.69 ± 1.54	.572
Platelet, mean ± SD	206.74 ± 58.08	220.48 ± 63.57	.024*
PSA, no(%)			<.001*
<10 ng/mL	49(25.4)	106(50.48)	
≥10 ng/mL	145(74.6)	104(49.52)	
Gleason Score, no. (%)			
Less or 6	36(18.6)		
7	67(34.5)		
8 or higher	91(46.9)		
ALT(IU/L), mean ± SD	20.08 ± 10.87	21.71 ± 13.21	.177
AST(IU/L), mean ± SD	20.09 ± 8.09	19.37 ± 8.08	.372
AST/ALT ratio, mean ± SD	1.13 ± 0.45	1.00 ± 0.35	.002*

Abbreviations: ALT, alanine transaminase; AST, aspartate aminotransferase; BMI, body mass index; NLR, neutrophil/lymphocyte ratio; PSA, prostate‐specific antigen.

* Statistically significant.

### 3.1 Predictive factors for the incidence risk of PCa

Since AST/ALT ratio tended to be related to the incidence risk of PCa, we performed multivariate binary logistic regression to investigate the independent predictive factors for the incidence risk of PCa. AST/ALT ratio, age, platelet were taken into consideration. As given in Table 2, AST/ALT ratio could independently predict the incidence risk of PCa (OR 1.043, 95%CI 1.014‐1.072, *P* = .003), together with age (OR 1.908, 95%CI 1.098‐3.317, *P* = .022). In addition, AST/ALT ratio, age, platelet, BMI, and other factors were not statistically significant in high‐risk prostate cancer and low‐risk prostate cancer in Table 3. In addition, we did not find statistical difference between AST/ALT ratio in high‐risk PCa patients (n = 121) and low‐risk PCa patients (n = 92) (OR 1.158, 95%CI 0.591‐2.269, *P* = .67). And also, we suggested ASL, ALT, or NLR might have no association with high‐ or low‐risk PCa. According to the cutoff value, patients with AST/ALT ratio < 1.155 were assigned to low ratio group, while patients with the ratio > 1.155 were classified as high ratio group. And we found that age and BMI may affect the ratio of AST/ALT in these two groups (p＜0.001). We also did not find significant association between AST/ALT ratio and the malignancy of PCa in Table 4.

**Table 2:** Risk factors for the incidence of PCa

		Univariate analysis			Multivariate analysis		
Variables		OR	95CI%	*P* value	OR	95CI%	*P* value	
AST/ALT ratio	(continuous)	2.313	1.337‐4.003	.002*	1.043	1.014‐1.072	.003*	
Age	(continuous)	1.054	1.027‐1.082	<.001*	1.908	1.098‐3.317	.022*	
Platelet	(continuous)	0.996	0.993‐1	.024*	0.998	0.994‐1.001	.181	
BMI	(continuous)	1.041	0.981‐1.105	.182				
Diabetes	(presence vs absence)	1.09	0.608‐1.954	.773				
Hypertension	(presence vs absence)	0.922	0.622‐1.367	.687				
Coronary heart disease	(presence vs absence)	0.649	0.384‐1.098	.107				
NLR	(continuous)	0.963	0.845‐1.098	.572				
ALT	(continuous)	0.989	0.972‐1.005	.177				
AST	(continuous)	1.011	0.987‐1.036	.372				

Abbreviations: ALT, alanine transaminase; AST, aspartate transaminase; BMI, body mass index; CI, confidence interval; NLR neutrophil/lymphocyte ratio; OR, odds ratio.

* Statistically significant.

**Table 3:** Comparison of risk factors between high‐risk PCa and low‐risk PCa

Variables		OR	95CI%	*P* value
AST/ALT	(continuous)	1.158	0.591‐2.269	.67
Age	(continuous)	0.981	0.905‐1.064	.649
Platelet	(continuous)	1.001	0.996‐1.006	.774
BMI	(continuous)	1.018	0.982‐1.055	.323
NLR	(continuous)	0.953	0.783‐1.161	.635
ALT	(continuous)	1.015	0.987‐1.044	.304
AST	(continuous)	1.02	0.981‐1.061	.312

Abbreviations: ALT, alanine transaminase; AST, aspartate transaminase; BMI, body mass index; CI, confidence interval, NLR, neutrophil/lymphocyte ratio; OR, odds ratio.

**Table 4:** Clinical and pathological characteristics of low or high AST/ALT

	Low AST/ALT ratio (n = 281)	High AST/ALT ratio (n = 123)	*P* value
Age(years) mean ± SD	67.08 ± 7.85	70.58 ± 7.54	<.001*
BMI, mean ± SD	25.01 ± 3.01	23.47 ± 3.08	<.001*
Diabetes, no. (%)			.039*
Absent	239 (85)	113 (91.9)	
Present	42 (15)	10 (8.1)	
Hypertension, no. (%)			.103
Absent	153 (55.4)	76 (61.8)	
Present	128 (45.6)	47 (38.2)	
Coronary heart disease, no. (%)			.482
Absent	233 (83)	103 (83.7)	
Present	48 (17)	20 (16.3)	
NLR, mean ± SD	2.63 ± 1.43	2.69 ± 1.65	.675
Platelet, mean ± SD	216.19 ± 60.68	208.63 ± 63.63	.255
PSA, no(%)			.075
<10 ng/mL	119 (42.3)	81 (66)	
≥10 ng/mL	162 (57.6)	42 (34)	
ALT(U/L), mean ± *SD*	24.16 ± 13.03	13.54 ± 4.416	<.001*
AST(U/L), mean ± *SD*	19.55 ± 7.89	20.12 ± 8.67	.514
PCa, no. (%)			<.001*
Yes	117 (41.6)	77 (62.6)	
No	164 (58.4)	46 (37.4)	
PCa, no. (%)			.519
Low risk	44 (37.6)	28 (36.4)	
High risk	73 (62.4)	49 (63.6)	

Abbreviations: ALT, alanine transaminase; AST, aspartate aminotransferase; BMI, body mass index; NLR, neutrophil/lymphocyte ratio; PCa, prostate cancer; PSA, prostate‐specific antigen.

* Statistically significant.

## 4 DISCUSSION

Our results showed that AST/ALT ratio played an independent role in predicting the incidence risk of PCa and could be used as a good biochemical indicator to predict the occurrence of PCa. Platelets could not be used as independent predictors to predict PCa, but might be an important factor in combination with other predictors to improve the accuracy of diagnosing PCa. As an important factor, age also played an important role in the prediction of PCa. AST and ALT are the main circulating enzymes in serum, mainly in liver cells. When hepatocellular are damaged or death, AST and ALT in cells are released into the blood, resulting in increased blood AST and ALT levels.^9^ Lionel et al reported their study in 2010 and demonstrated that there was a significant association between ALT and pathological Gleason sum ≥ 7(4 + 3) cancer. And they also found that there was no significant association between AST or ALT with other adverse pathological features or biochemical recurrence after radical prostatectomy.^10^ In their study, the authors concluded that serum AST and ALT levels may be associated with a higher Gleason score, but not with biochemical recurrence. The discovery of this phenomenon, we guessed that liver cell damage indirectly affects the metabolism of androgen in the liver, so that the level of androgen in the blood changes, which may lead to the occurrence and development of PCa.^10^ But our results showed that AST and ALT levels were not directly related to the malignancy of PCa. The reason for this contradiction may be due to the small number of people we include, or the different race or living environment.

Recently studies in renal cell carcinoma^11^ and upper tract urothelial carcinoma^12^ demonstrated that AST/ALT ratio played a significantly prognostic role in urological tumor. Mona et al demonstrated that AST is expressed in different tissue types throughout the body, while ALT is mostly expressed in hepatocytes and has tissue specificity.^13^ We assumed that when certain organs or tissues of the body were abnormal or damaged, AST levels might change at different levels, resulting in a change of AST/ALT ratio. Zoppini et al reported that high AST/ALT ratio may indicated systemic variations.^7^ These alterations might suggest that AST/ALT ratio may increase due to tumor cells. Wang et al showed that AST/ALT ratio as an independent risk factor for biochemical recurrence‐free survival in PCa patients underwent radical prostatectomy. And they also thought higher AST/ALT ratio was found to be related to greater Gleason Score. Although the AST/ALT ratio is still controversial in predicting the prognosis of PCa, the current clinical researches found that the change of AST/ALT ratio is indeed related to the pathophysiology of PCa.

## 5 CONCLUSION

Although there were few studies on AST/ALT ratio in the risk of PCa, and there was no relevant research to find out the impact of AST/ALT ratio on the incidence risk of PCa, but through the current researches, we could still understand the serum AST/ALT ratio was a correlation between the onset of PCa. The specific mechanism was still unclear, but we suspected that the ability of the liver to metabolize testosterone affected changes in systemic hormone levels, which may affect the growth and proliferation of tumor cells. Through our research, we can further understand the potential significance of AST/ALT ratio in PCa, and it is more likely to be an important biochemical indicator in the future to predict the occurrence of PCa and the prognosis of PCa. We still needed a lot of clinical studies and long‐term follow‐up to clarify the specific relationship between AST/ALT ratio and the incidence risk of PCa.

## CONFLICT OF INTEREST

The authors declare no conflict of interest.

## AUTHORS’ CONTRIBUTIONS

JZ and RL were involved in conception and design, revising it for intellectual content, and gave final approval of the completed article. ZH and SM were involved in extraction of data, and drafting the article. All authors read and approved the final manuscript.

## ETHICAL STATEMENT

All procedures performed in studies involving human participants were in accordance with the ethical standards of the institution and/or national research committee. This article does not contain any studies with animals performed by any of the authors.

## CONSENT FOR PUBLICATION

Not applicable.

## Data Availability

All data generated or analyzed during this study are included in this published article.
